# Extensive dysregulations of oligodendrocytic and astrocytic connexins are associated with disease progression in an amyotrophic lateral sclerosis mouse model

**DOI:** 10.1186/1742-2094-11-42

**Published:** 2014-03-06

**Authors:** Yiwen Cui, Katsuhisa Masaki, Ryo Yamasaki, Shihoko Imamura, Satoshi O Suzuki, Shintaro Hayashi, Shinya Sato, Yuko Nagara, Mami F Kawamura, Jun-ichi Kira

**Affiliations:** 1 Department of Neurology, Neurological Institute, Graduate School of Medical Sciences, Kyushu University, 3-1-1 Maidashi, Higashi-ku, Fukuoka 812-8582, Japan; 2 Department of Neurological Therapeutics, Neurological Institute, Graduate School of Medical Sciences, Kyushu University, Fukuoka, Japan; 3 Department of Neuropathology, Neurological Institute, Graduate School of Medical Sciences, Kyushu University, Fukuoka, Japan

**Keywords:** amyotrophic lateral sclerosis, astrocyte, connexin, gap junction, oligodendrocyte, superoxide dismutase 1

## Abstract

**Background:**

Non-cell-autonomous motor neuronal death is suggested in a mutant Cu/Zn superoxide dismutase 1 (mSOD1)-mediated amyotrophic lateral sclerosis (ALS) model, in which glial cells play significant roles in disease progression. Connexins (Cxs) form homotypic or heterotypic gap junctions (GJs) and allow direct intercellular communications among nervous tissue cells. The role of Cxs in motor neuron disease has never been investigated; therefore, we aimed to evaluate alterations of Cxs in mSOD1-transgenic (mSOD1-Tg) mice in comparison with their non-transgenic (non-Tg) littermates at the same ages.

**Methods:**

We pathologically evaluated temporal changes to astrocytic Cx43/Cx30 and oligodendrocytic Cx47/Cx32 immunoreactivities at presymptomatic, disease-progressive, and end stages, relative to aquaporin-4 (AQP4), glial fibrillary acidic protein (GFAP), excitatory amino acid transporter-2 (EAAT2), myelin-oligodendrocyte glycoprotein (MOG), and Nogo-A immunoreactivities, and observed neuronal loss by NeuN and neurofilament immunostaining, and microglial response by Iba-1 immunostaining. We also performed quantitative immunoblotting and real-time PCR analyses for Cxs.

**Results:**

The mSOD1-Tg mice showed neuronal and axonal loss in the anterior horns of the lumbar spinal cord accompanied by increased activation of microglia compared with non-Tg mice at the disease-progressive and end stages. Expression patterns of Cxs were not different between mSOD1-Tg and non-Tg mice at the presymptomatic stage, but immunoreactivities for GFAP, Cx43, Cx30 and AQP4 were increased in the anterior horns of mSOD1-Tg mice at the disease-progressive and end stages. By contrast, Cx47 and Cx32 immunoreactivities were markedly diminished in Nogo-A-positive oligodendrocytes in the anterior horns of mSOD1-Tg mice at the disease-progressive and end stages, especially in oligodendrocytes showing SOD1 accumulation. EAAT2 immunoreactivity was also diminished in the anterior horns of mSOD1-Tg mice at the disease-progressive and end stages. Quantitative immunoblotting revealed a significant reduction in Cx47 and Cx32 protein levels in mSOD1-Tg mice at the disease-progressive and end stages. The levels of Cx47 and Cx32 mRNAs were also decreased at these stages.

**Conclusions:**

Our findings indicate that oligodendrocytic and astrocytic GJ proteins in the anterior horns of spinal cord in mSOD1-Tg mice are profoundly affected at the disease-progressive and end stages, where disruption of GJs among glial cells may exacerbate motor neuronal death.

## Background

Amyotrophic lateral sclerosis (ALS) is a chronic fatal neurodegenerative disease characterized by progressive motor paralysis due to degeneration of upper and lower motor neurons in the brain and spinal cord. Although the detailed pathogenic mechanisms are still unknown, the contribution of glial cells to the pathogenesis of ALS has become a focus of basic and translational research. Astrocytes and microglia have been extensively investigated; evidence from studies using selective gene excision [1,2] or bone-marrow grafting [3] has shown that mutant SOD1-derived alterations in either microglia or astrocytes accelerate later disease progression in mice. In human beings, it was reported that astrocytes and neural progenitor cells derived from postmortem spinal cord of sporadic ALS and familial ALS patients shared a common non-cell autonomous toxicity, as shown by the selective killing of motor neurons in a co-culture model system [4].

By contrast, the contribution of oligodendrocytes has been relatively ignored until lately. Oligodendrocytes, the myelin-forming cells of the central nervous system (CNS), maintain long-term axonal integrity and provide an energy supply to neurons [5]. Recently, Lee *et al.*[6] and Philips *et al.*[7] demonstrated that oligodendrocytes abundantly express monocarboxylate transporter 1 (MCT1), a lactate transporter, and that disruption of this transporter induces axon damage and neuron loss in a mouse model of ALS. Expression of MCT1 was also diminished in patients with ALS [6], suggesting that oligodendrocyte dysfunction might contribute to the pathogenesis of ALS. However, it remains unknown whether these glial cells surrounding motor neurons can communicate normally with each other to maintain homeostasis in this condition.

In this study, we focused on connexins (Cxs); these form homotypic or heterotypic gap junctions (GJs) between adjacent astrocytes, or between astrocytes and oligodendrocytes. Gap junctions appose two cells and form channels for direct intercellular communication, through which intracellular second messengers, such as calcium ions and small molecules, are exchanged. Astrocytes mainly express Cx43 and Cx30, while oligodendrocytes express Cx32 and Cx47 [8]. In the CNS, glucose and lactate can diffuse through astrocytes via GJs into neighboring astrocytes [9,10]. Astrocytes transfer lactate or glucose (or both) to oligodendrocytes through heterocellular GJ channels between them [9]. Although few studies have focused on Cxs in motor neuron disease, Díaz-Amarilla *et al.*[11] described how astrocytes with an aberrant phenotype isolated from symptomatic rats carrying a *SOD1* gene mutation showed augmented Cx43 immunoreactivity but lacked glutamate transporter 1 (GLT-1), also known as excitatory amino acid transporter-2 (EAAT2). Because these aberrant astrocytes specifically induced motor neuron death in a co-culture system, these authors proposed that upregulation of Cx43 in aberrant astrocytes might trigger glial activation and induce excitotoxic degeneration of motor neurons [11]. Here, we demonstrate that the levels of oligodendrocytic Cx47 and Cx32 are markedly diminished in the anterior horns of spinal cords from mSOD1-Tg mice, suggesting that disruption of the glial syncytium due to alteration of Cx expression might contribute to the progression of motor neuron disease.

## Methods

### Mice and tissue preparation

Transgenic mice carrying human G93A mSOD1 (B6SJL-TgN (SOD1-G93A) 1Gur/J; 002726) were acquired from the Jackson Laboratory (Bar Harbor, ME, USA) and bred in the Center of Biological Research, Graduate School of Medical Sciences, Kyushu University. These mice were crossed with female mice with a C57BL/6 background for at least four generations. Transgenic offspring were genotyped by PCR of DNA obtained from tail biopsies. These animals exhibited a predictable disease onset at about 16 weeks after birth, with leg tremor and decreased stride and muscle strength, and died at almost 20 weeks after birth. In this study, female and male mice were sacrificed at presymptomatic (aged 12 weeks, *n* = 11), disease-progressive (aged 18 weeks, *n* = 13) and end (aged 20 weeks, *n* = 13) stages. This study was approved by the Recombinant DNA Experiment Safety Committee, Graduate School of Medical Sciences, Kyushu University. Animals were handled in conformity with the guidelines for the care and use of laboratory animals of our institution. In all experiments, mSOD1-Tg mice were sacrificed together with age-matched non-transgenic (non-Tg) littermates.

### Immunohistochemistry

To obtain spinal cord tissues, mice (*n* = 5 to 7 per group) were deeply anesthetized and perfused transcardially with PBS, and then with 4% paraformaldehyde in 0.1 M phosphate buffer. The spinal cords were carefully dissected to identify the lumbar segments. The tissues were fixed in 10% buffered formalin and processed into paraffin sections (5 μm thick). We performed immunohistochemistry using an indirect immunoperoxidase method. Deparaffinized sections were hydrated in ethanol and then incubated with 0.3% hydrogen peroxide in absolute methanol for 30 min at room temperature to inhibit endogenous peroxidase. After rinsing with tap water, the sections were washed using Tris–HCl with 0.1% Triton X-100 for 5 min, twice, and then with Tris–HCl for 5 min. After this pretreatment, the sections were incubated with a primary antibody diluted in a mixture of 5% normal goat serum, 50 mM Tris-HCl (pH 7.6) and 1% BSA at 4°C overnight. After rinsing, sections were subjected to labeling with either a streptavidin–biotin complex or an enhanced indirect immunoperoxidase method using Envision (DakoCytomation, Glostrup, Denmark). The colored reaction product was developed using a 3,3'-diaminobenzidine tetrahydrochloride hydrate solution. Sections were counterstained with hematoxylin. The primary antibodies used for immunohistochemistry are listed in Table 1. Cx43, Cx30, glial fibrillary acidic protein (GFAP), aquaporin-4 (AQP4) and EAAT2 were used as astrocyte markers. Cx32, Cx47, myelin-oligodendrocyte glycoprotein (MOG), and Nogo-A were employed as oligodendrocyte or myelin markers.

**Table 1:** Antibodies used for immunohistochemistry

**Antigen**	**Type**	**Dilution**	**Antigen retrieval**	**Source**
Astrocyte				
Cx43	Rabbit polyclonal	1:1000	Not done	Abcam, Cambridge, UK
Cx30	Rabbit polyclonal	1:100	Autoclave, 10 mM citrate buffer	Sigma Aldrich, St Louis, USA
AQP4	Rabbit polyclonal	1:500	Not done	Santa Cruz Biotechnology, California, USA
GFAP	Rabbit polyclonal	1:1000	Not done	DakoCytomation, Glostrup, Denmark
EAAT2	Mouse monoclonal	1:50	Not done	Novocastra, Newcastle upon Tyne, UK
Oligodendrocyte or myelin				
Cx32	Mouse monoclonal	1:200	Autoclave, 10 mM citrate buffer	Life Technologies, California, USA
Cx47	Mouse monoclonal	1:200	Autoclave, 10 mM citrate buffer	Life Technologies, California, USA
MOG	Rabbit polyclonal	1:1000	Not done	Sigma Aldrich, St Louis, USA
Nogo-A	Rabbit polyclonal	1:400	Not done	Santa Cruz Biotechnology, California, USA
CC1	Mouse monoclonal	1:1000	Autoclave, 10 mM citrate buffer	Abcam, Cambridge, UK
Microglia				
Iba-1	Rabbit polyclonal	1:1000	Not done	Wako, Osaka, Japan
Neuron				
NeuN	Mouse monoclonal	1:100	Not done	Merck Millipore, Darmstadt, Germany
Axon				
Neurofilament	Mouse monoclonal	1:100	Not done	DakoCytomation, Glostrup, Denmark
Superoxide dismutase 1				
SOD1	Rabbit polyclonal	1:100	Autoclave, 10 mM citrate buffer	Abcam, Cambridge, UK
Apoptotic cell				
Cleaved caspase-3	Rabbit polyclonal	1:1000	Autoclave, 10 mM citrate buffer	Cell Signaling Technology, MA, USA

AQP4, aquaporin-4; Cx, connexin; EAAT2, excitatory amino acid transporter-2; GFAP, glial fibrillary acidic protein; Iba-1, ionized calcium-binding adapter molecule 1; MOG, myelin-oligodendrocyte glycoprotein; SOD1, superoxide dismutase 1.

### Indirect immunofluorescence and confocal laser microscopy

Using the same set of paraffin sections, double immunofluorescence staining was performed with the following combinations of antibodies: rabbit polyclonal anti-Nogo-A and mouse monoclonal anti-Cx47; rabbit polyclonal anti-Nogo-A and mouse monoclonal anti-Cx32; rabbit polyclonal anti-SOD1 and mouse monoclonal anti-Cx47. All sections were deparaffinized in xylene and rehydrated through an ethanol gradient. Sections were then incubated with primary antibodies overnight at 4°C. After rinsing, sections were incubated with an Alexa 488-conjugated goat anti-mouse immunoglobulin G (IgG) and an Alexa 546-conjugated goat anti-rabbit IgG (Invitrogen), and then counterstained with 4',6-diamidino-2-phenylindole (DAPI). Images were captured using a confocal laser microscope system (Nikon A1, Nikon, Tokyo, Japan). We used the sequential multiple fluorescence scanning mode to avoid non-specific overlap of colors, and captured all pictures under the same conditions of magnification, laser intensity, gain and offset values, and pinhole setting.

### Quantitative immunoblot analysis

Mice (*n* = 3 to 5 per group) were transcardially perfused using phosphate-buffered saline and spinal cords were dissected. Samples were collected in lysis matrix D tubes (MP Biomedicals, Solon, OH) and immersed in the mixture of radioimmunoprecipitation assay (RIPA) buffer and 0.5% sodium dodecyl sulfate. Samples were homogenized using a rapidly oscillating BioMasher instrument. Tissue samples were kept on ice for 1 h and centrifuged at 4°C for 10 min at 13,000*g*. Supernatants were collected and analyzed for protein contents using the BSA protein assay (ThermoScientific, Rockford, IL). Eight micrograms of tissue (or 2 μg for analysis of GFAP) was loaded onto a 12% Mini-PROTEAN TGX gel (Bio-Rad, Hercules, CA). After electrophoresis, samples were blotted onto a polyvinyldifluoride membrane using a wet blotting apparatus (Bio-Rad). After blotting, gels were incubated in 3% skimmed milk or 3% BSA in Tris-buffered saline containing 0.1% Tween-20 (TBS-T). After washing the membrane, the membrane was incubated with primary antibodies (Table 2) overnight at 4°C. Membranes were washed in TBS-T and incubated with horseradish peroxidase-conjugated secondary antibodies. After incubation, membranes were washed and visualized by enhanced chemiluminescence (ECL Prime, GE Healthcare Bio-Sciences AB, Uppsala, Sweden). The band intensity was measured using the ChemiDoc XRS system (Bio-Rad) and normalized to that of the glyceraldehyde-3-phosphate dehydrogenase (GAPDH) band.

**Table 2:** Antibodies used for Western blotting

**Antigen**	**Type**	**Dilution**	**Source**
Astrocyte			
Cx43	Rabbit polyclonal	1:1000	Abcam, Cambridge, UK
Cx30	Rabbit polyclonal	1:200	Sigma Aldrich, St Louis, USA
GFAP	Rabbit polyclonal	1:1000	DakoCytomation, Glostrup, Denmark
EAAT2	Mouse monoclonal	1:50	Novocastra, Newcastle upon Tyne, UK
Oligodendrocyte/myelin			
Cx32	Rabbit polyclonal	1:250	Life Technologies, California, USA
Cx47	Mouse monoclonal	1:200	Life Technologies, California, USA
MOG	Rabbit polyclonal	1:1000	Sigma Aldrich, St Louis, USA
Housekeeping protein			
GAPDH	Mouse monoclonal	1:10000	Santa Cruz Biotechnology, California, USA

Cx, connexin; EAAT2, excitatory amino acid transporter-2; GAPDH, glyceraldehyde 3-phosphate dehydrogenase.

GFAP, glial fibrillary acidic protein; MOG, myelin-oligodendrocyte glycoprotein.

### RNA extraction and real-time reverse-transcription (RT)-PCR

Spinal cord tissues of non-Tg (*n* = 3 per group) and mSOD1-Tg mice (*n* = 3 per group) were removed and homogenized using a rapidly oscillating bio masher. Total RNA was extracted using an RNeasy Mini kit (Qiagen, Hilden, Germany) according to the manufacturer’s protocol. Total RNA (20 ng) was converted to cDNA by reverse transcription using a Transcriptor First Strand cDNA Synthesis Kit (Roche Applied Science, Mannheim, Germany). A primer pair designed against GAPDH was used as an internal control. The expression levels of the genes encoding Cx32 (*Gjb1*), Cx47 (*Gjc2*), Cx30 (*Gjb6*), and Cx43 (*Gja1*) were assessed by quantitative real-time PCR (qPCR) analysis performed under the following conditions: 55°C for 2 min and 95°C for 10 min, followed by 50 cycles at 95°C for 15 s and 60°C for 1 min. Real-time PCR was performed using Taq-Man® Gene Expression Assays (Cx32: Mm01950058-s1; Cx43: Mm01179639-s1; Cx47: Mm00519131-s1; Cx30: Mm00433661-s1) and a 7500 Real-Time PCR System (Applied Biosystems, Carlsbad, CA). The threshold cycle (Ct) of target genes was normalized to that of GAPDH. Expression levels of mRNA in mSOD1-Tg mice were calculated after normalizing cycle thresholds against GAPDH and are presented as the fold induction value (2^−ΔΔCt^) relative to non-Tg mice (mean ± standard deviation).

### Quantitative analysis of oligodendrocytes

Oligodendrocytes were labeled with anti-Nogo-A antibody. We counted cells within one side of the anterior horn that was defined as a gray matter area separated by vertical and horizontal lines from the central canal. Four to six slices per mouse at 20 weeks of age were randomly selected from 5 μm-thick lumbar spinal cord paraffin sections, and labeled oligodendrocytes were counted manually by an examiner blinded to the experimental conditions. Cell numbers on one side of the anterior horn were averaged, and the mean cell numbers in each mouse (*n* = 4) were analyzed statistically, as described next.

### Statistical analysis

Data were analyzed using Microsoft Excel software and are expressed as means ± standard error of the mean. Significance was assessed using Student’s *t* test, and *P* values less than 0.05 were considered statistically significant.

## Results

### General pathology and microglial activation in the anterior horns of the spinal cords of non-Tg and mSOD1-Tg mice

There was no difference in the numbers of neurons and axons in the anterior horns of spinal cords between non-Tg and mSOD1-Tg mice at 12 weeks of age. However, in mSOD1-Tg mice at 18 and 20 weeks of age, the number of NeuN-positive motor neurons and the immunoreactivity for neurofilament were decreased in the anterior horns compared with those in non-Tg mice (Additional file 1: Figure S1A–D). Expression of neurofilament was also diminished in the anterior roots of mSOD1-Tg mice (Additional file 1: Figure S1C,D). Ramified microglia with fine processes were observed in the anterior horns of non-Tg mice, whereas numerous morphologically-activated microglia were present in the anterior horns of mSOD1-Tg mice at 18 and 20 weeks of age (Additional file 1: Figure S1E,F). The number of activated microglia in the anterior horns of mSOD1-Tg mice was stage-dependently increased (Additional file 2: Figure S2A-C).

### Expression of astrocytic and oligodendrocytic Cxs in the anterior horns of the spinal cords of non-Tg mice

**Figure 1:**
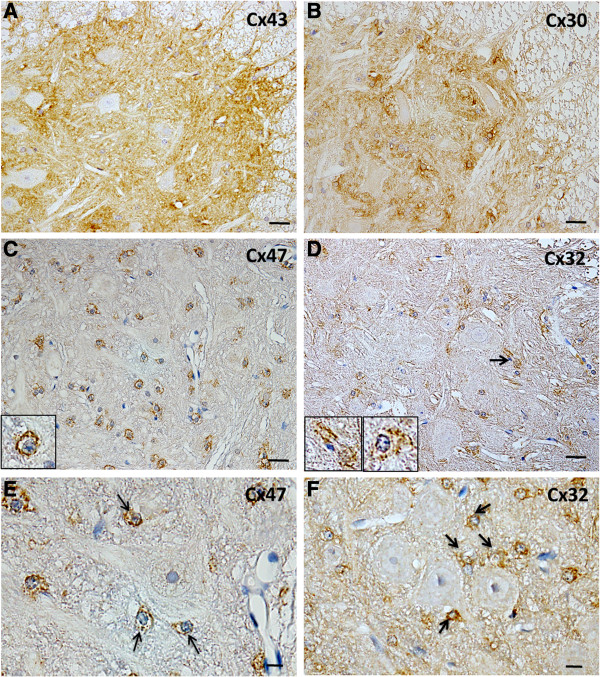
**Normal expression patterns of Cxs in the anterior horn of the spinal cord. (A,B)** Immunoreactivities for Cx43 and Cx30 are diffusely expressed in the anterior horn of the spinal cord from a non-Tg mouse at 18 weeks of age, showing neuropil staining. **(C,D)** Immunoreactivities for Cx47 and Cx32 are highlighted around the oligodendrocyte somata in the anterior horns of the spinal cord (insets) and Cx32 is also expressed along myelin sheaths (**D**, arrow). **(E,F)** At higher magnification, immunoreactivities for Cx47 and Cx32 are observed along the surface of satellite oligodendrocytes adjacent to motor neurons (arrows). Scale bar; 20 μm **(A-D)**, 10 μm **(E,F)**.

Cx43 and Cx30 were diffusely expressed in the anterior horns of spinal cord of non-Tg mice at 18 weeks of age, with staining of astrocytic fine processes (Figure 1A,B). Strong immunoreactivities for Cx47 and Cx32 were seen around the oligodendrocyte somata, and Cx32 was expressed on myelin fibers in the anterior horns of the spinal cord (Figure 1C,D). Satellite oligodendrocytes close to motor neurons also expressed Cx47 and Cx32 (Figure 1E,F).

### Astrocytic changes in the anterior horns of the spinal cords of mSOD1-Tg mice

**Figure 2:**
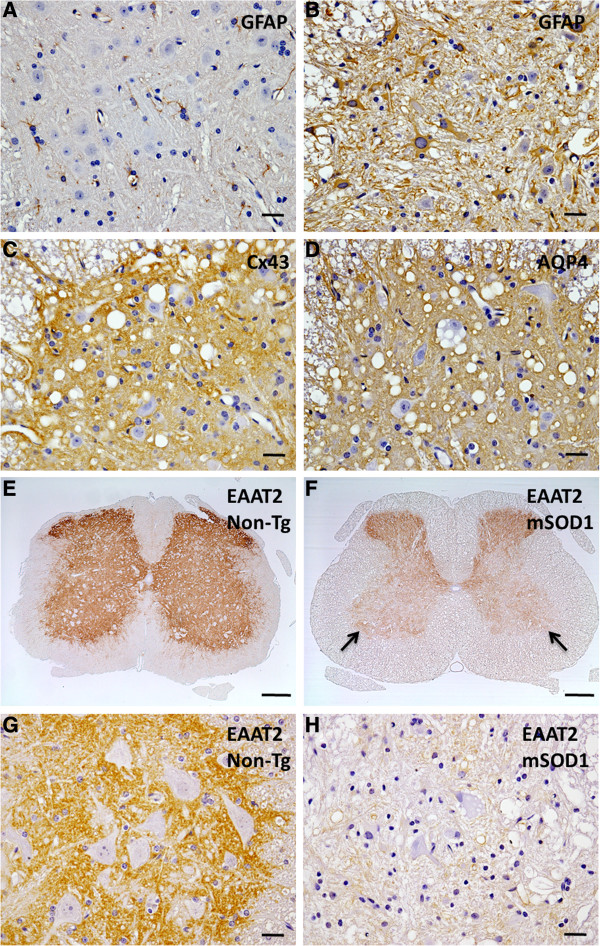
**Astrogliotic changes in the anterior horns of spinal cords from mSOD1-Tg mice. (A)** GFAP immunostaining reveals normal-shaped astrocytic cytoplasm and fine processes in the anterior horns of spinal cords from non-Tg mice, whereas **(B)** numerous gemistocytes are visible in the anterior horns of spinal cord from mSOD1-Tg mice at 18 weeks of age. **(C,D)** Immunoreactivities for Cx43 and AQP4 are increased in the anterior horns of the spinal cords of mSOD1-Tg mice. **(E,F)** By contrast, expressions of EAAT2 are downregulated in the anterior horns of mSOD1-Tg mice compared with non-Tg mice (arrows). **(G,H)** At higher magnification, immunoreactivity for EAAT2 is markedly diminished in the anterior horns of mSOD1-Tg mice compared with non-Tg mice. Scale bar; 20 μm **(A-D,G,H)**, 200 μm **(E,F)**.

There was no difference in the morphology of astrocytes in the anterior horns of spinal cords between non-Tg and mSOD1-Tg mice at 12 weeks of age. However, in the mSOD1-Tg mice at 18 and 20 weeks of age, immunoreactivity for GFAP was stage-dependently upregulated and numerous hypertrophic astrocytes existed in the anterior horns compared with non-Tg mice (Figure 2A,B and Additional file 2: Figure S2D–F). Levels of Cx43 and AQP4 were also upregulated in the anterior horns of the spinal cords of all mSOD1-Tg mice examined (Figure 2C,D). Immunoreactivity for Cx30 was preserved in the anterior horns of mSOD1-Tg mice (data not shown). By contrast, immunoreactivity for EAAT2 was diminished in the anterior horns of mSOD1-Tg mice compared with non-Tg mice (Figure 2E-H). Downregulation of EAAT2 in the anterior horns was observed in three of seven mSOD1-Tg mice (42.9%) at 18 weeks of age and two of five mSOD1-Tg mice (40.0%) at 20 weeks of age. There was no significant alteration of any astrocytic or oligodendrocytic markers in mSOD1-Tg mice compared with non-Tg mice at 12 weeks of age.

### Oligodendrocytic changes in the anterior horns of the spinal cords of mSOD1-Tg mice

**Figure 3:**
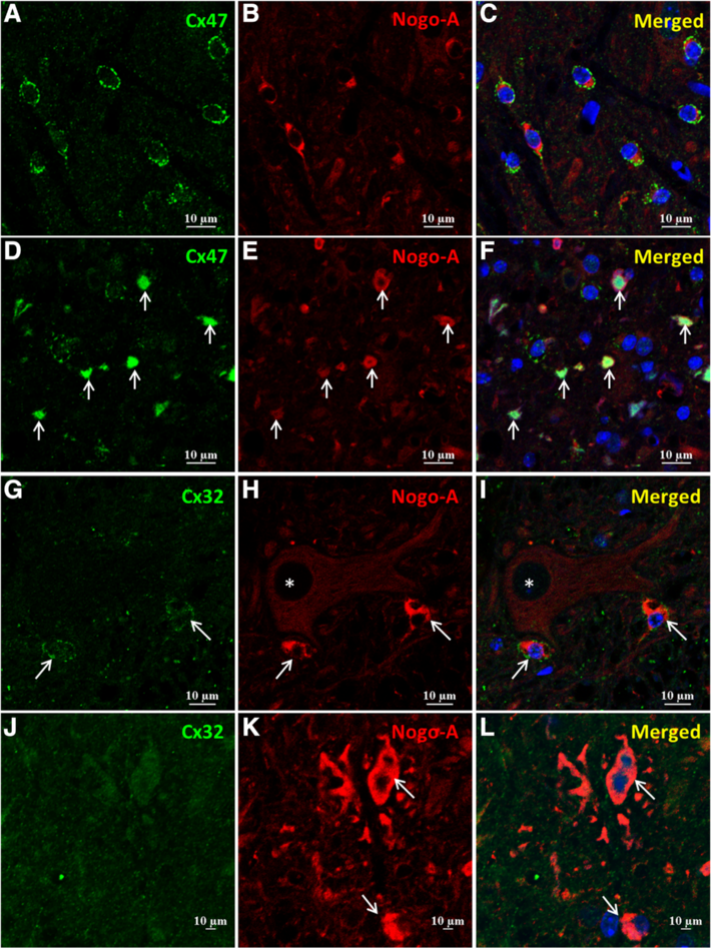
**Decreased membranous expressions of Cx47 and Cx32 in mSOD1-Tg mice at 20 weeks of age. (A-C)** Immunoreactivity for Cx47 is normally present at the surface membrane of oligodendrocytes in non-Tg mice at 20 weeks of age, while **(D-F)** membranous staining of Cx47 is markedly decreased and internalized to the cytoplasm of oligodendrocytes in the anterior horns of mSOD1-Tg mice at the same age (arrows). **(G-I)** Immunoreactivity for Cx32 is also mainly localized at the membrane of oligodendrocyte somata in non-Tg mice (arrows), whereas **(J-L)** Cx32 expression in oligodendrocyte somata is diminished in the anterior horns of mSOD1-Tg mice at 20 weeks of age (arrows). **(H,I)** Asterisks show motor neurons adjacent to oligodendrocytes. **(E,K)** Oligodendrocytes in the anterior horns of mSOD1-Tg mice demonstrate an abnormal-shaped morphology. The nucleus is stained with DAPI (**A-L**, blue). Scale bar; 10 μm **(A-L)**.

Double immunofluorescence for Cx32/Cx47 and Nogo-A revealed that immunoreactivity for Cx47 was localized to the membrane of Nogo-A-positive oligodendrocytes in the anterior horns of non-Tg mice at 20 weeks of age (Figure 3A-C). By contrast, in all mSOD1-Tg mice at 18 and 20 weeks of age, membranous expression of Cx47 in oligodendrocytes was diminished and Cx47 was internalized to the cytoplasm in the anterior horns (Figure 3D-F). Like Cx47, expression of Cx32 was also mainly observed in the surface membrane of anterior horn oligodendrocytes (Figure 3G-I) in non-Tg mice. In all mSOD1-Tg mice at 18 and 20 weeks of age, expression of Cx32 at the oligodendrocytic membrane was decreased in the anterior horns; however, internalization of Cx32 was not detectable (Figure 3J-L). Immunostaining for Nogo-A revealed abnormal-shaped oligodendrocytes in the anterior horns of mSOD1-Tg mice at 18 and 20 weeks of age (Figure 3E,K). Membrane Cx47 and Cx32 were obviously downregulated in these abnormal-shaped oligodendrocytes. We also performed immunochemistry for Cx47 and Cx32 using an indirect immunoperoxidase method in mSOD1-Tg mice at 20 weeks of age (Additional file 2: Figure S2A,B). In agreement with these-mentioned results, immunoreactivity for Cx47 was observed in the cytoplasm (Additional file 3: Figure S3A) and that for Cx32 at the membranes of oligodendrocytes was decreased in mSOD1-Tg mice compared with non-Tg mice (Additional file 3: Figure S3B). We counted the Nogo-A-positive differentiated oligodendrocytes in the anterior horns of the spinal cords of non-Tg and mSOD1-Tg mice at 20 weeks of age (Additional file 4: Figure S4). There was no significant difference in the total number of oligodendrocytes in mSOD1-Tg mice compared with non-Tg mice. Immunoreactivity for MOG in the anterior horns was not different between the two groups at all stages (data not shown). There was no significant alteration of any astrocytic and oligodendrocytic markers in mSOD1-Tg mice as compared with non-Tg mice at 12 weeks of age.

### Quantitative immunoblot analysis of Cxs in non-Tg mice and mSOD1-Tg mice

**Figure 4:**
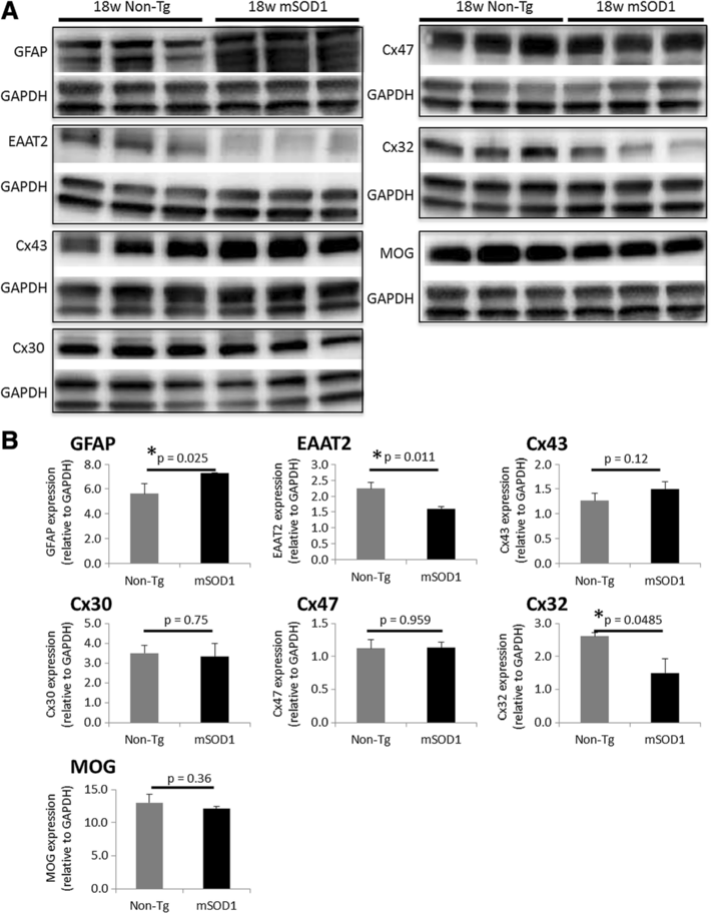
**Quantitative immunoblot analysis of Cxs in mSOD1-Tg and non-Tg mice at 18 weeks of age. (A)** Representative images of GFAP, EAAT2, Cx43, Cx30, Cx47, Cx32, and MOG immunoblots obtained from mSOD1-Tg mice and non-Tg mice (*n* = 3 per group). GAPDH blots for loading controls are shown under each protein blot. **(B)** Results of quantitative analysis for each protein as indicated. Expression levels of EAAT2 and Cx32 are significantly decreased in mSOD1-Tg mice compared with non-Tg mice. By contrast, the level of GFAP expression is increased in mSOD1-Tg mice than non-Tg mice. The expression levels of the other markers, including Cx43, Cx30, and Cx47, are not significantly different between the two groups.

**Figure 5:**
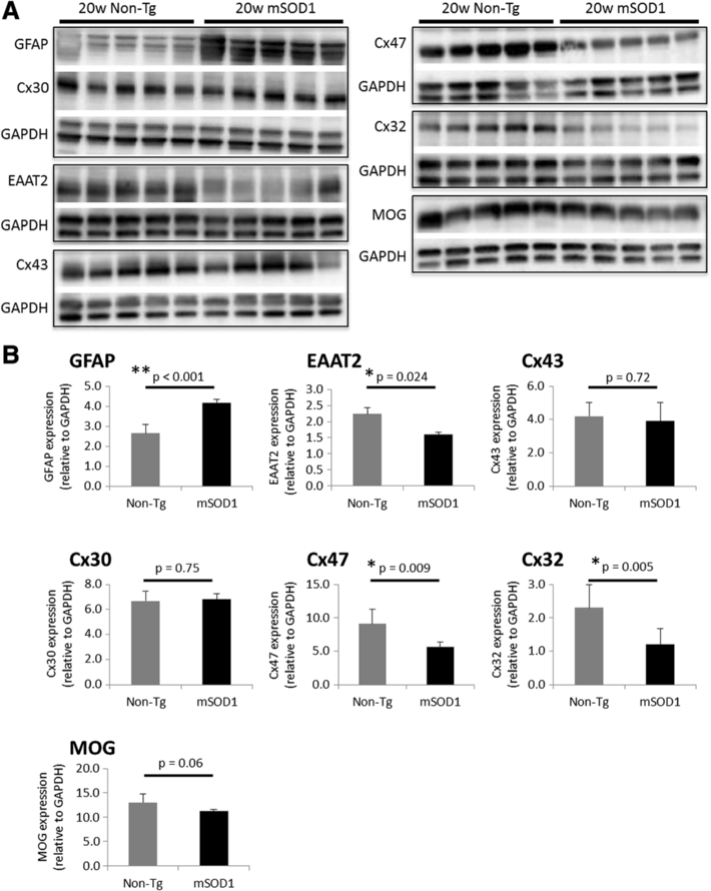
**Quantitative immunoblot analysis of Cxs in mSOD1-Tg and non-Tg mice at 20 weeks of age. (A)** Representative images of GFAP, EAAT2, Cx43, Cx30, Cx32, Cx47, and MOG immunoblots obtained from mSOD1-Tg mice and non-Tg mice (*n* = 5 per group) at 20 weeks of age. GAPDH blots for loading controls are shown under each protein blot. **(B)** Results of quantitative analysis for each astrocyte or oligodendrocyte marker. The expression levels of Cx47, Cx32, and EAAT2 are significantly decreased in mSOD1-Tg mice than in non-Tg mice. By contrast, the expression levels of GFAP are significantly increased in mSOD1-Tg mice as compared with non-Tg mice. Cx43, Cx30, and MOG expressions are not significantly different between mSOD1-Tg and non-Tg mice.

We performed immunoblot analyses of Cx proteins at different time points in non-Tg and mSOD1-Tg mice. There was no significant change in any markers of astrocytes or oligodendrocytes in mSOD1-Tg mice compared with non-Tg mice at 12 weeks of age (Additional file 5: Figure S5A,B). At 18 weeks of age, levels of EAAT2 and Cx32 were significantly decreased in mSOD1-Tg mice compared with non-Tg mice (for EAAT2; *P* = 0.011, for Cx32; *P* = 0.048), whereas the level of GFAP was increased in mSOD1-Tg mice compared with non-Tg mice (*P* = 0.025). No statistically significant differences in the levels of Cx43, Cx30, or Cx47 were observed between the two groups (Figure 4A,B). At 20 weeks of age, the levels of Cx47, Cx32, and EAAT2 were significantly reduced in mSOD1-Tg mice compared with those in non-Tg mice (for Cx47, *P* = 0.009; for Cx32, *P* = 0.005; for EAAT2, *P* = 0.024) whereas the levels of Cx43, Cx30, and MOG were not significantly altered between mSOD1-Tg and non-Tg mice. The level of GFAP was increased in mSOD1-Tg mice compared with non-Tg mice at 20 weeks of age (*P* < 0.001) (Figure 5A,B).

### Quantitative real-time PCR analysis of Cxs in non-Tg mice and mSOD1-Tg mice

**Figure 6:**
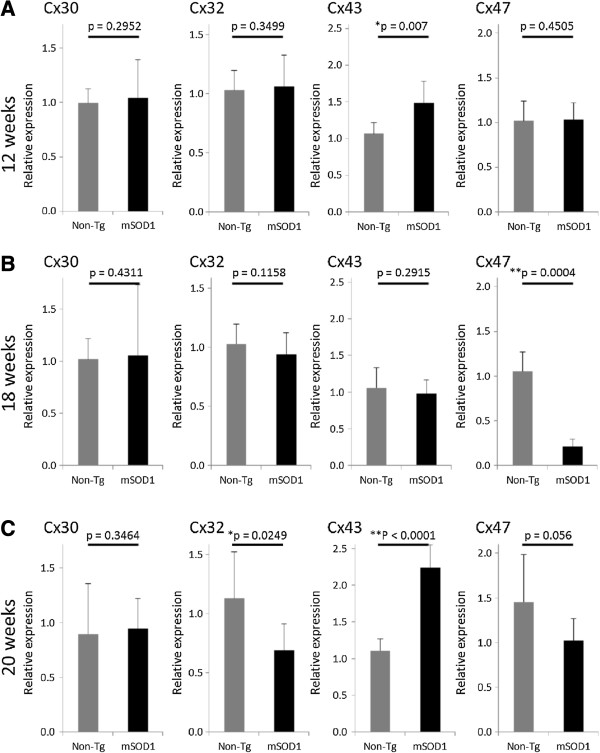
**Comparison of Cx mRNA expression levels in mSOD1-Tg and non-Tg mice by quantitative real-time PCR. (A)** At 12 weeks of age, the relative expression level of Cx43 mRNA in mSOD1-Tg mice is significantly increased compared with that in non-Tg mice. **(B)** At 18 weeks of age, the expression level of Cx47 mRNA is significantly decreased in mSOD1-Tg mice. **(C)** At 20 weeks of age, the expression level of Cx43 mRNA is significantly increased in mSOD1 mice, whereas that of Cx32 is significantly decreased. In addition, the expression level of Cx47 mRNA shows a decreased tendency in mSOD1-Tg mice. (*P < 0.05, **P < 0.001).

We measured the mRNA expression levels of Cxs in non-Tg and mSOD1-Tg mice at 12, 18, and 20 weeks of age. The mRNA level of Cx43 was significantly increased in mSOD1-Tg mice compared with non-Tg mice at 12 weeks of age (*P* = 0.007), while there were no significant differences in the mRNA levels of Cx30, Cx32, and Cx47 between the two groups (Figure 6A). At 18 weeks of age, the mRNA level of Cx47 was significantly reduced in mSOD1-Tg mice compared with that in non-Tg mice (*P* = 0.0004), while there were no significant differences in the levels of Cx30, Cx32, and Cx43 (Figure 6B). At 20 weeks of age, the mRNA level of Cx32 was significantly decreased in mSOD1-Tg mice compared with that in non-Tg mice (*P* = 0.0249), whereas the level of Cx43 was significantly increased in the former compared with that in the latter (*P* < 0.0001). Moreover, in mSOD1-Tg mice, the level of mRNA for Cx47 showed a tendency toward a reduction compared with that in non-Tg mice at 20 weeks of age (*P* = 0.056) (Figure 6C).

### Overexpression of SOD1 in the anterior horn oligodendrocytes of mSOD1-Tg mice

**Figure 7:**
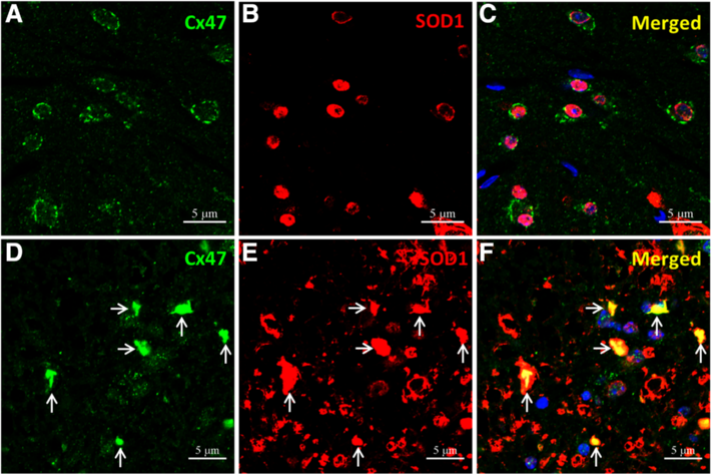
**Overexpression of SOD1 in oligodendrocytes of mSOD1-Tg mice. (A-C)** Immunoreactivity for SOD1 is observed in the nuclei and cytoplasm of oligodendrocytes in the anterior horns of non-Tg mice at 20 weeks of age and immunoreactivity for Cx47 is preserved at the surface of oligodendrocytes. **(D-F)** However, SOD1 is markedly accumulated in neurons and astrocytes in the anterior horns of mSOD1-Tg mice at 20 weeks of age and accumulation of SOD1 was also observed in oligodendrocytes with Cx47-immunopositivity in their cytoplasm (arrows). Membranous staining of Cx47 is not visible in abnormal-shaped oligodendrocytes. The nucleus is stained with DAPI (**A**-**F** blue). Scale bar; 5 μm **(A-F)**.

To elucidate the mechanism by which mSOD1 affects expression of Cx47 and Cx32, we performed double immunofluorescence staining for mSOD1 and Cx47/Cx32 in non-Tg and mSOD1-Tg mice. At 20 weeks of age, immunopositivity for SOD1 was subtle in the nuclei and cytoplasm of oligodendrocytes in the anterior horns of non-Tg mice (Figure 7A-C). By contrast, expression of SOD1 was markedly upregulated in the anterior horns of mSOD1-Tg mice. The cytoplasmic accumulation of SOD1 was also observed in dysmorphic Cx47-positive oligodendrocytes in mSOD1-Tg mice, and membranous staining of Cx47 was no longer observed on these abnormal oligodendrocytes (Figure 7D-F).

### Expression of cleaved caspase-3 in the anterior horn oligodendrocytes of mSOD1-Tg mice

**Figure 8:**
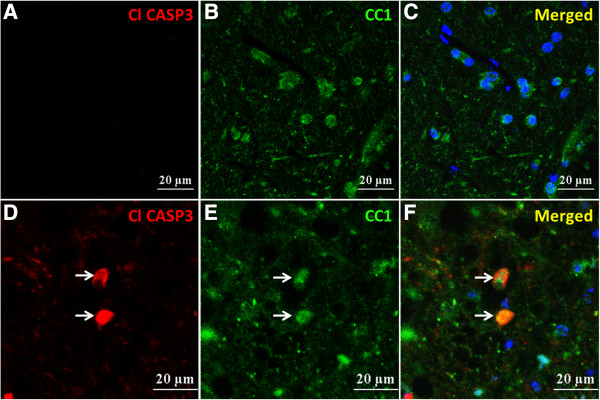
**Cleaved caspase-3 expression in the anterior horn oligodendrocytes of mSOD1-Tg mice.** Double immunostaining for cleaved caspase-3 and CC1 was performed in the anterior horns of spinal cords from non-Tg and mSOD1-Tg mice at 20 weeks of age. **(A-C)** Immunoreactivity for cleaved caspase-3 is never observed in the anterior horn of non-Tg mice. **(D-F)** Cleaved caspase-3 expression is detected in a small number of CC1-positive mature oligodendrocytes in the anterior horns of mSOD1-Tg mice (arrows). Scale bar; 20 μm **(A-F)**.

To clarify the possible causes of the morphological changes in oligodendrocytes, we performed double immunostaining for CC1 (an oligodendrocyte marker) and cleaved caspase-3, a marker of apoptotic cell death. A small number of cleaved caspase-3-positive oligodendrocytes were present in the anterior horns of mSOD1-Tg mice at 18 and 20 weeks of age, whereas such changes were never observed in non-Tg mice (Figure 8).

## Discussion

In this study, by performing detailed immunohistochemical analyses supplemented by quantitative immunoblotting and real-time PCR analyses on the expression of Cxs at different stages of disease in mSOD1-Tg mice, we disclosed the following novel findings. (1) Although there was no difference in MOG expression between non-Tg and mSOD1-Tg mice at all stages of illness, the surface membrane levels of oligodendrocytic Cx47 and Cx32 based on immunohistochemistry were markedly diminished in the anterior horns where immunostaining for Nogo-A revealed the emergence of abnormal-shaped oligodendrocytes at the disease-progressive and end stages. Quantitative immunoblotting and real-time PCR analyses also confirmed a decrease in Cx47 and Cx32 expression levels in mSOD1-Tg mice at the advanced disease stage. (2) By contrast, immunoreactivity for astrocytic Cx43 was extensively upregulated in the anterior horns of mSOD1-Tg mice at the progressive and end stages. Real-time PCR analysis suggested that, even at the presymptomatic stages, astrocytic Cx43 expression levels increased. This change coincided with upregulation of GFAP and downregulation of EAAT2, as shown by immunohistochemistry and quantitative immunoblotting. These findings are summarized in Table 3. Glial connexin changes have also been found in other neurological disorders, such as multiple sclerosis, Alzheimer’s disease, Parkinson’s disease, and epilepsy. In multiple sclerosis and its animal model, experimental autoimmune encephalomyelitis, expression of oligodendrocytic Cx32 and Cx47 was markedly downregulated in chronic demyelinating plaques of the white matter [12-14]. In Alzheimer’s disease, upregulation of Cx43 was detected in the cerebral cortical astrocytes in amyloid β-containing plaques [15,16]. The 1-methyl-4-phenyl-1,2,3,6-tetrahydropyridine (MPTP) mouse model of Parkinson’s disease showed upregulation of Cx43 in the striatum [17]. Upregulation of Cx43 and Cx32 has also been detected in different types of epilepsy in human beings [18,19]. Although various CNS pathological conditions could cause alteration of glial connexins, extensive loss of oligodendrocytic Cx47/32 in the anterior horns appears to be specific for mSOD1-Tg mouse spinal cord.

**Table 3:** Summary of oligodendrocytic and astrocytic changes in the anterior horns of mSOD1-Tg mice

	**12 weeks of age (presymptomatic stage)**				**18 weeks of age (disease-progressive stage)**				**20 weeks of age (end stage)**		
	**Immunohistochemistry (*n *** **= 5)**	**qIB (*n *** **= 3)**	**qPCR (*n *** **= 3)**		**Immunohistochemistry ( *n *** **= 7)**	**qIB (*n *** **= 3)**	**qPCR (*n *** **= 3)**		**Immunohistochemistry (*n *** **= 5)**	**qIB (*n *** **= 5)**	**qPCR (*n *** **= 3)**
Cx32	Unchanged	Unchanged	Unchanged		Decreased	Decreased	Unchanged		Decreased	Decreased	Decreased
Cx47	Unchanged	Unchanged	Unchanged		Decreased (internalized)	Unchanged	Decreased		Decreased (internalized)	Decreased	Unchanged
Cx30	Unchanged	Unchanged	Unchanged		Unchanged	Unchanged	Unchanged		Unchanged	Unchanged	Unchanged
Cx43	Unchanged	Unchanged	Increased		Increased	Unchanged	Unchanged		Increased	Unchanged	Increased
GFAP	Unchanged	Unchanged	Not done		Increased	Increased	Not done		Increased	Increased	Not done
EAAT2	Unchanged	Unchanged	Not done		Decreased (3/7)	Decreased	Not done		Decreased (2/5)	Decreased	Not done
MOG	Unchanged	Unchanged	Not done		Unchanged	Unchanged	Not done		Unchanged	Unchanged	Not done

Cx, connexin; EAAT2, excitatory amino acid transporter-2; GFAP, glial fibrillary acidic protein; MOG, myelin-oligodendrocyte glycoprotein; qIB, quantitative immunoblot; qPCR, quantitative real-time polymerase chain reaction.

The level of Cx32 protein in oligodendrocytes was reduced even at the disease-progressive stage, while transcription of Cx32 was unchanged at the disease-progressive stage and first decreased at the end stage, suggesting that Cx32 degradation might be enhanced. Membranous expression of Cx47 started to decrease at the disease-progressive stage, which is attributable to deceased transcription of Cx47 and defective transport from the cytosol to the surface membrane or increased internalization from the membrane to the cytosol, or both. These Cx changes were especially evident in the abnormal-shaped oligodendrocytes with accumulated SOD1 at the anterior horns. Kang *et al.*[20] demonstrated that selective removal of mutant SOD1 from oligodendrocytes delayed disease onset and prolonged survival in mSOD1-Tg mice, suggesting that mutations in the *SOD1* gene could accelerate disease progression by directly impairing the function of oligodendrocytes. In the gray matter oligodendrocytes, non-Tg mice had subtle expression of SOD1 in the nuclei and cytoplasm, as previously reported [21]. By contrast, we observed accumulation of SOD1 in mSOD1-Tg mice, which is in accord with the report of Stieber *et al.*[22] that aggregates of mutant SOD1 protein appeared not only in neurons and astrocytes but also in oligodendrocytes and their periaxonal processes by immuno-electron microscopy. Overexpression of SOD1 in the gray matter oligodendrocytes coincided with diminished membranous expression of Cx47 and Cx32, which are usually expressed in the oligodendrocyte somata and their proximal processes [23]. Mutant SOD1 protein is said to acquire toxic functions, most likely through mutation-driven conformational changes [24,25]. Thus, although we could not directly demonstrate a causative relationship between mutant SOD1 and Cx pathology, decreased expression of Cx47 and Cx32 in the abnormal-shaped oligodendrocytes could be in part attributable to abnormal SOD1 accumulation. In addition, we showed stage-dependent progression of astrogliosis and microglial activation in the anterior horns of mSOD1-Tg mice. Thus, reactive astrocytes and activated microglia may also partially contribute to the oligodendrocyte pathology in mSOD1-Tg mice.

Some cells in mSOD1-Tg mice had typical Cx47 gap junction plaques on their surfaces but were Nogo-A negative, as opposed to the Nogo-A-positive cells with cytoplasmic Cx47 signals. Nogo-A is considered to be a reliable marker for mature oligodendrocytes, like other markers such as CC-1 and CNPase [26]. However, Kuhlmann *et al.*[26] reported that *in-situ* hybridization for proteolipid protein mRNA was more sensitive for detection of oligodendrocytes than immunostaining for Nogo-A and CC-1. Thus, we speculate that the Nogo-A-negative, Cx47-positive cells may represent residual oligodendrocytes. Another possibility is that the Nogo-A-negative-Cx47-positive cells may be oligodendrocyte precursor cells (OPCs), because Philips *et al.*[7] recently reported that OPCs show increased proliferation and differentiation in the spinal cords of mSOD1-Tg mice.

Although the precise role of the gray matter oligodendrocytes still remains unknown, previous studies have suggested that satellite (perineuronal) oligodendrocytes might provide metabolic support to the associating neurons, rather than regulate their synaptic transmission [27]. Recently, it was reported that the anterior horn oligodendrocytes in mSOD1-Tg mice downregulated the lactate transporter MCT1 and that disruption of the glycolytic pathway yielded insufficient energy to support neuronal survival [6,7]. In the CNS, astrocyte-astrocyte and astrocyte-oligodendrocyte Cx gap junction channels allow intercellular trafficking of glucose and its metabolites among the glial syncytium [9,10]. In normal conditions, oligodendrocytes can import glucose through glucose transporter 1 (GLUT1) and Cx junctions for glycolysis. Glycolysis can yield sufficient ATP to support oligodendrocyte survival while lactate, an aerobic glycolysis product, can be transferred to axons via MCT1 and used as an energy source for axonal activity [5]. Thus, we consider that loss of membranous Cx47 and Cx32 in oligodendrocytes may lead to insufficient glucose supply, and subsequently contribute to oligodendrocytic damage and accelerate motor neuron death through energy failure in mSOD1 ALS model mice. Indeed, we detected upregulation of cleaved caspase-3 in mature oligodendrocytes in the anterior horns of mSOD1-Tg mice, suggesting that these cells become apoptotic. Such caspase activation was never seen in non-Tg mice. Our notion is supported by the finding that mice lacking Cx32 and Cx47 in oligodendrocytes showed not only severe demyelination or oligodendrocyte cell death, but also axonal loss [28].

Concerning astrocytic Cx involvement, because oligodendrocytic Cx47 and Cx32 were downregulated in mSOD1-Tg mice at the disease-progressive and end stages, the remaining astrocytic Cx43 would form hemichannels. Moreover, Orellana *et al.* reported an *in-vitro* study showing that activated microglia can release pro-inflammatory cytokines, which increase astrocytic Cx43 hemichannel activities [9]. Thus, numerous activated microglia present in the gray matter of mSOD1 ALS model mice at the advanced stages could also increase astrocytic Cx43 hemichannel activities. Then, neurotoxic substances such as glutamate could be released from the disconnected Cx hemichannels into the extracellular space [9,29,30], thereby damaging adjacent motor neurons [31]. Glutamate and ATP released via Cx43 hemichannels facilitate opening of pannexin 1 (Panx1) hemichannels in neurons [32-34]. Opened Panx1 hemichannels could contribute to neuronal death through Ca^2+^ entry and activation of intracellular neurotoxic cascades [34]. Connexin hemichannel blockers have been shown to reduce inflammation and improve functional recovery after spinal cord injury, brain abscess, and ischemia [35-37]. Takeuchi *et al.*[38] also found that the blockade of GJs by a novel blood-brain barrier-permeable gap junction blocker successfully suppressed disease progression in ALS model mice. Accordingly, astrocytic Cx43 hemichannels might also contribute to motor neuron death in ALS model mice and could be a potential therapeutic target in future.

## Conclusions

For the first time, we described in detail the stage-dependent alteration of glial Cxs in ALS model mice. Our findings indicate that oligodendrocytic and astrocytic GJs are affected in the gray matter of mSOD1-Tg mice, where disruption of GJs among glial cells might contribute to acceleration of motor neuron disease progression.

## Abbreviations

ALS: amyotrophic lateral sclerosis; AQP4: aquaporin-4; BSA: bovine serum albumin; CNS: central nervous system; Ct: threshold cycle; Cx: connexin; DAPI: 4',6-diamidino-2-phenylindole; EAAT2: excitatory amino acid transporter-2; GAPDH: glyceraldehyde-3-phosphate dehydrogenase; GFAP: glial fibrillary acidic protein; GJ: gap junction; GLT-1: glutamate transporter 1; Iba-1: ionized calcium-binding adapter molecule 1; IgG: immunoglobulin G; MCT1: monocarboxylate transport 1; MOG: myelin-oligodendrocyte glycoprotein; MPTP: 1-methyl-4-phenyl-1,2,3,6-tetrahydropyridine; OPC: oligodendrocyte precursor cells; Panx1: pennexin 1; PBS: phosphate-buffered saline; PCR: polymerase chain reaction; qPCR: quantitative real-time polymerase chain reaction; RIPA: radioimmunoprecipitation assay; RT-PCR: reverse-transcription polymerase chain reaction; SOD1: superoxide dismutase 1; Tg: transgenic.

## Competing interests

The authors declare that they have no competing interests.

## Authors’ contributions

YWC contributed to the design of the study, performed immunohistochemistry, immunofluorescence, immunoblotting, quantification and analysis, and prepared the draft of the manuscript. KM and RY contributed to the conceptual design and preparation of the manuscript. SI and SH contributed to the quantitative real-time PCR analysis, SOS contributed to the analysis of results, SS contributed to the preparation of immunoblotting, YN and MFK contributed to the preparation of animal experiments, JK contributed to the design of the study and to the preparation of the manuscript. All authors have read and approved the final version of the manuscript.

## Supplementary Material

Additional file 1: Figure S1 Neuronal and microglial pathology in non-Tg and mSOD1-Tg mice. **(A,C)** In the anterior horns of spinal cord in non-Tg mice at 18 weeks of age, NeuN-positive neurons and neurofilament-positive axons are abundantly observed, whereas **(B,D)** in mSOD1-Tg mice, the numbers of neurons and axons are markedly decreased in the anterior horns and in the anterior roots (arrows in **C**,**D**). **(E,F)** Immunostaining for Iba-1 shows that numerous ramified microglia are present in the anterior horns of non-Tg mice, whereas activated, hypertrophic microglia are predominant in those of mSOD1-Tg mice at 18 weeks of age. Scale bar; 20 μm **(A-D)**, 10 μm **(E,F)**. Click here for file

Additional file 2: Figure S2 Stage-dependent progression of astrogliosis and microglial activation in the anterior horns of mSOD1-Tg mice. Immunostaining for Iba-1 and GFAP was performed in mSOD1-Tg mice at 12 weeks (**A** and **D**, respectively), 18 weeks (**B** and **E**, respectively), and 20 weeks (**C** and **F**, respectively) of age. Immunostaining for Iba-1 reveals stage-dependent activation of microglia in the anterior horns of mSOD1-Tg mice **(A-C)**. Immunostaining for GFAP shows stage-dependent progression of astrogliosis in the anterior horns of mSOD1-Tg mice **(D-F)**. Scale bar; 20 μm **(A-F)**. Click here for file

Additional file 3: Figure S3 Decreased membranous staining of Cx47 and Cx32 in the anterior horn oligodendrocytes of mSOD1-Tg mice. **(A,C)** In the anterior horns of the spinal cord in mSOD1-Tg mice at 20 weeks of age, immunoreactivities for Cx47 and Cx32 at the surface membrane of oligodendrocytes are markedly diminished. **(B)** At higher magnification, immunoreactivity for Cx47 is found in the oligodendrocytic cytoplasm, whereas **(D)** immunoreactivity for Cx32 is not detectable in the oligodendrocyte somata. Scale bar; 20 μm **(A,C)**, 10 μm **(B,D)**. Click here for file

Additional file 4: Figure S4 Oligodendrocyte cell number in mSOD1-Tg mice and non-Tg mice at 20 weeks of age. There is no significant difference in total Nogo-A-positive oligodendrocyte cell number between mSOD1-Tg and non-Tg mice at 20 weeks of age (*n* = 4). Data represent means ± standard error of the mean. Click here for file

Additional file 5: Figure S5 Quantitative immunoblot analysis of Cxs in mSOD1-Tg mice and non-Tg mice at 12 weeks of age. **(A)** Representative images of GFAP, EAAT2, Cx43, Cx30, Cx47, Cx32 and MOG immunoblots obtained from mSOD1-Tg mice and non-Tg mice (*n* = 3 per group). GAPDH blots for loading controls are shown under each protein blot. **(B)** Results of quantitative analysis for each protein. There is no statistically significant difference between mSOD1-Tg and non-Tg mice for any markers of astrocytes or oligodendrocytes. Click here for file
