# Supplementary figures and images for: Extensive dysregulations of oligodendrocytic and astrocytic connexins are associated with disease progression in an amyotrophic lateral sclerosis mouse model
Source: J Neuroinflammation. 2014 Mar 6;11:42. doi: 10.1186/1742-2094-11-42 (PMC4016493; doi:10.1186/1742-2094-11-42)

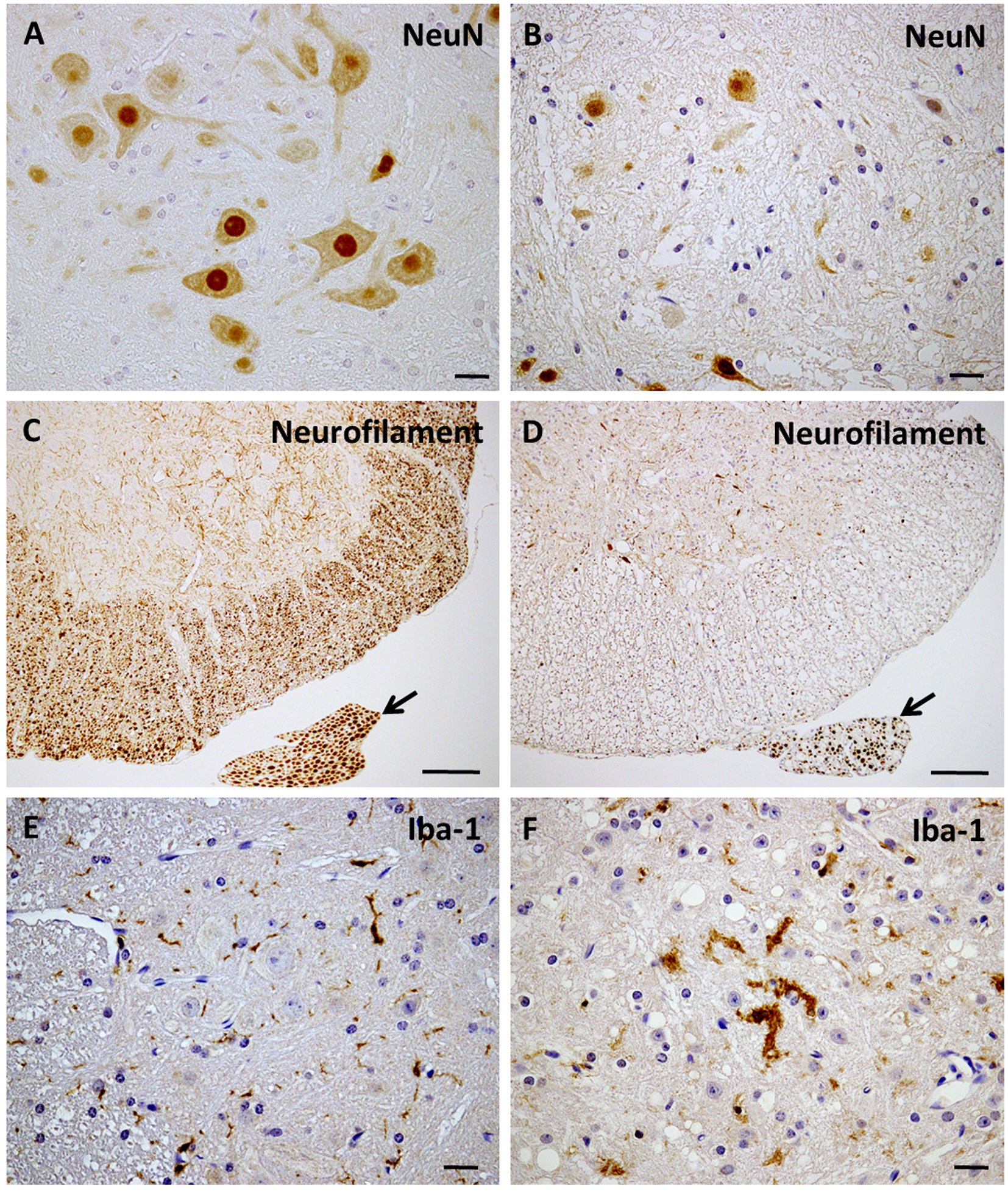

Supplement: Additional file 1: Figure S1 — Neuronal and microglial pathology in non-Tg and mSOD1-Tg mice. (A,C) In the anterior horns of spinal cord in non-Tg mice at 18 weeks of age, NeuN-positive neurons and neurofilament-positive axons are abundantly observed, whereas (B,D) in mSOD1-Tg mice, the numbers of neurons and axons are markedly decreased in the anterior horns and in the anterior roots (arrows in C,D). (E,F) Immunostaining for Iba-1 shows that numerous ramified microglia are present in the anterior horns of non-Tg mice, whereas activated, hypertrophic microglia are predominant in those of mSOD1-Tg mice at 18 weeks of age. Scale bar; 20 μm (A-D), 10 μm (E,F). [file 1742-2094-11-42-S1.tiff]

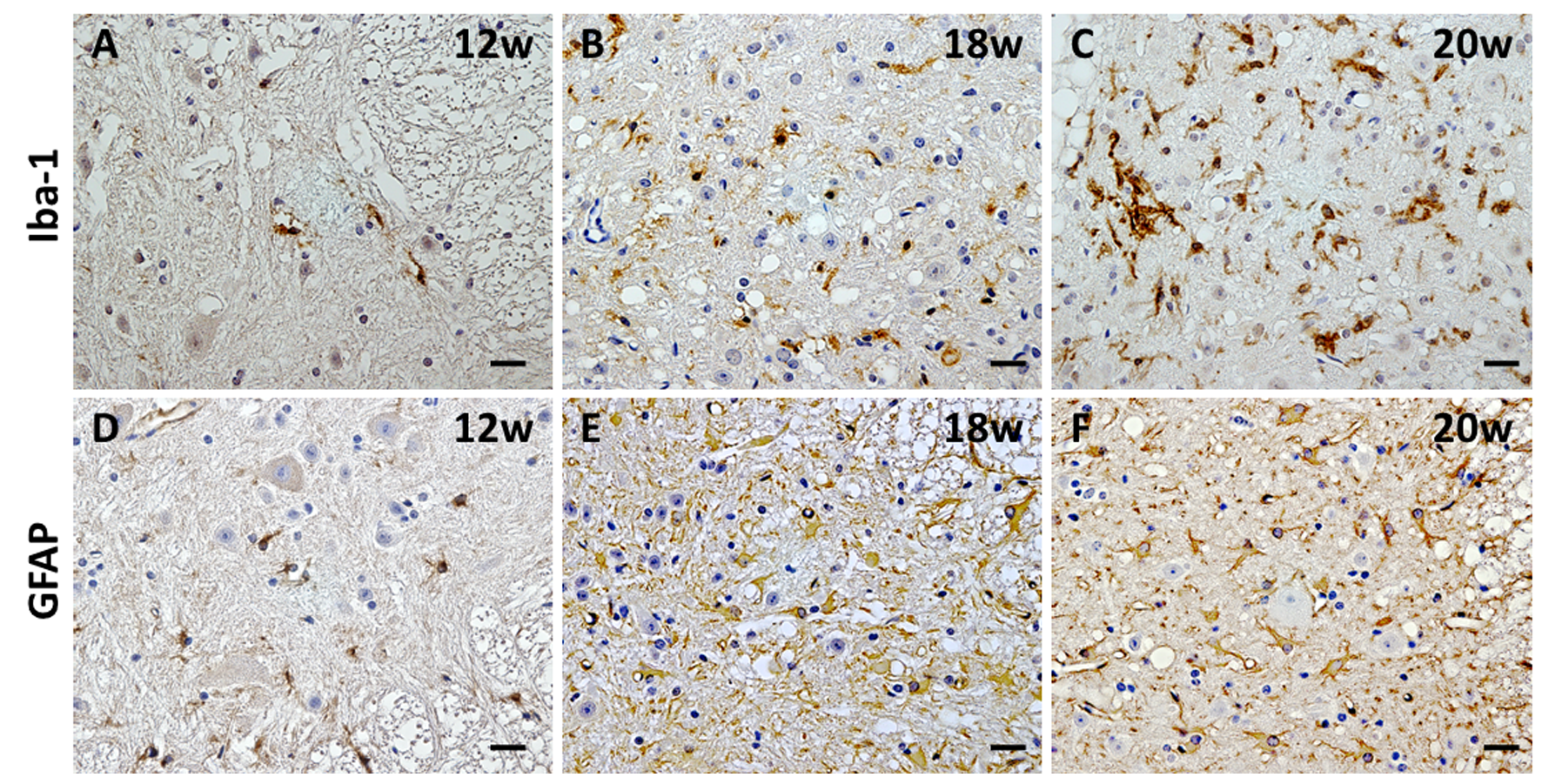

Supplement: Additional file 2: Figure S2 — Stage-dependent progression of astrogliosis and microglial activation in the anterior horns of mSOD1-Tg mice. Immunostaining for Iba-1 and GFAP was performed in mSOD1-Tg mice at 12 weeks (A and D, respectively), 18 weeks (B and E, respectively), and 20 weeks (C and F, respectively) of age. Immunostaining for Iba-1 reveals stage-dependent activation of microglia in the anterior horns of mSOD1-Tg mice (A-C). Immunostaining for GFAP shows stage-dependent progression of astrogliosis in the anterior horns of mSOD1-Tg mice (D-F). Scale bar; 20 μm (A-F). [file 1742-2094-11-42-S2.tiff]

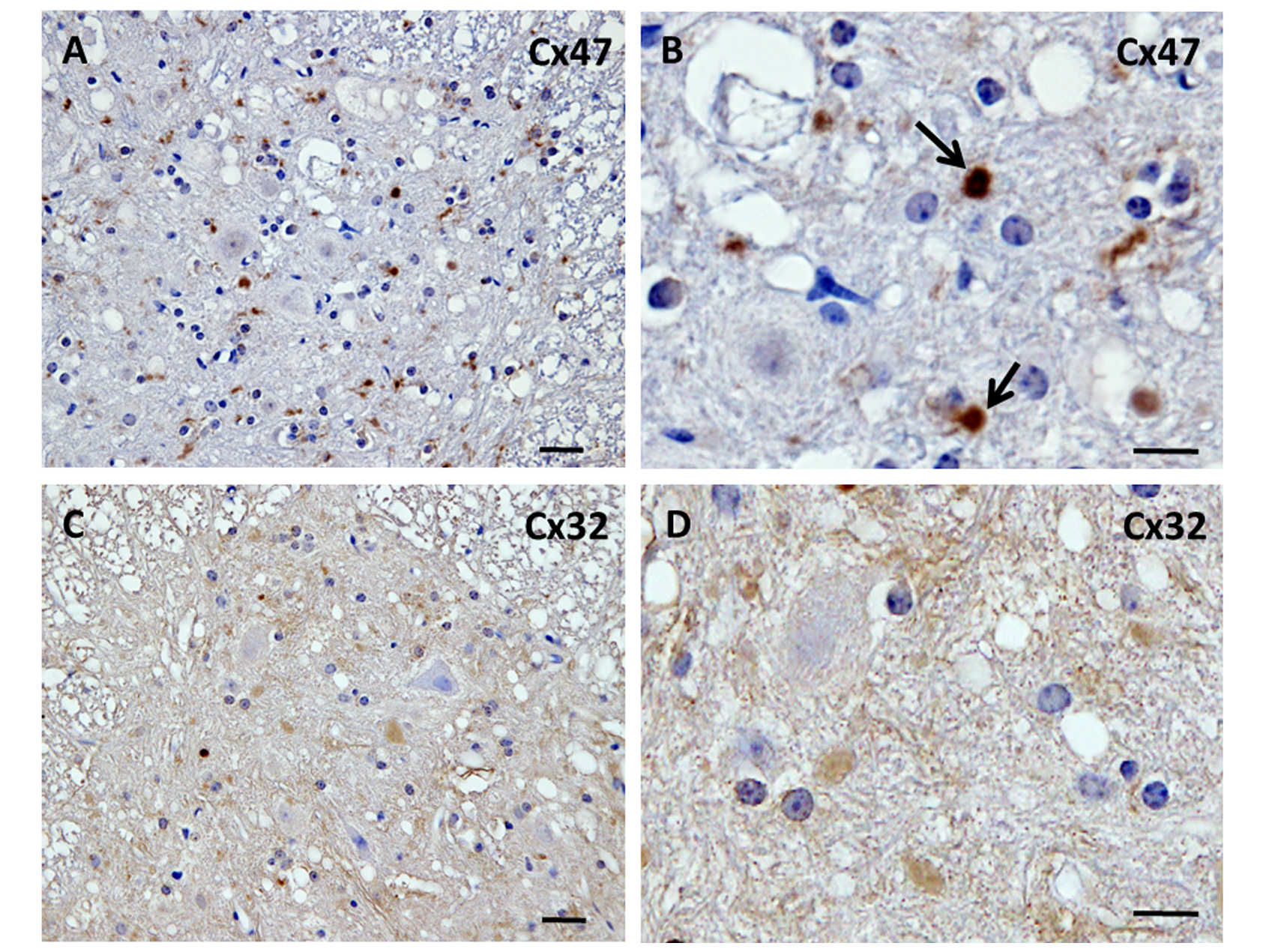

Supplement: Additional file 3: Figure S3 — Decreased membranous staining of Cx47 and Cx32 in the anterior horn oligodendrocytes of mSOD1-Tg mice. (A,C) In the anterior horns of the spinal cord in mSOD1-Tg mice at 20 weeks of age, immunoreactivities for Cx47 and Cx32 at the surface membrane of oligodendrocytes are markedly diminished. (B) At higher magnification, immunoreactivity for Cx47 is found in the oligodendrocytic cytoplasm, whereas (D) immunoreactivity for Cx32 is not detectable in the oligodendrocyte somata. Scale bar; 20 μm (A,C), 10 μm (B,D). [file 1742-2094-11-42-S3.tiff]

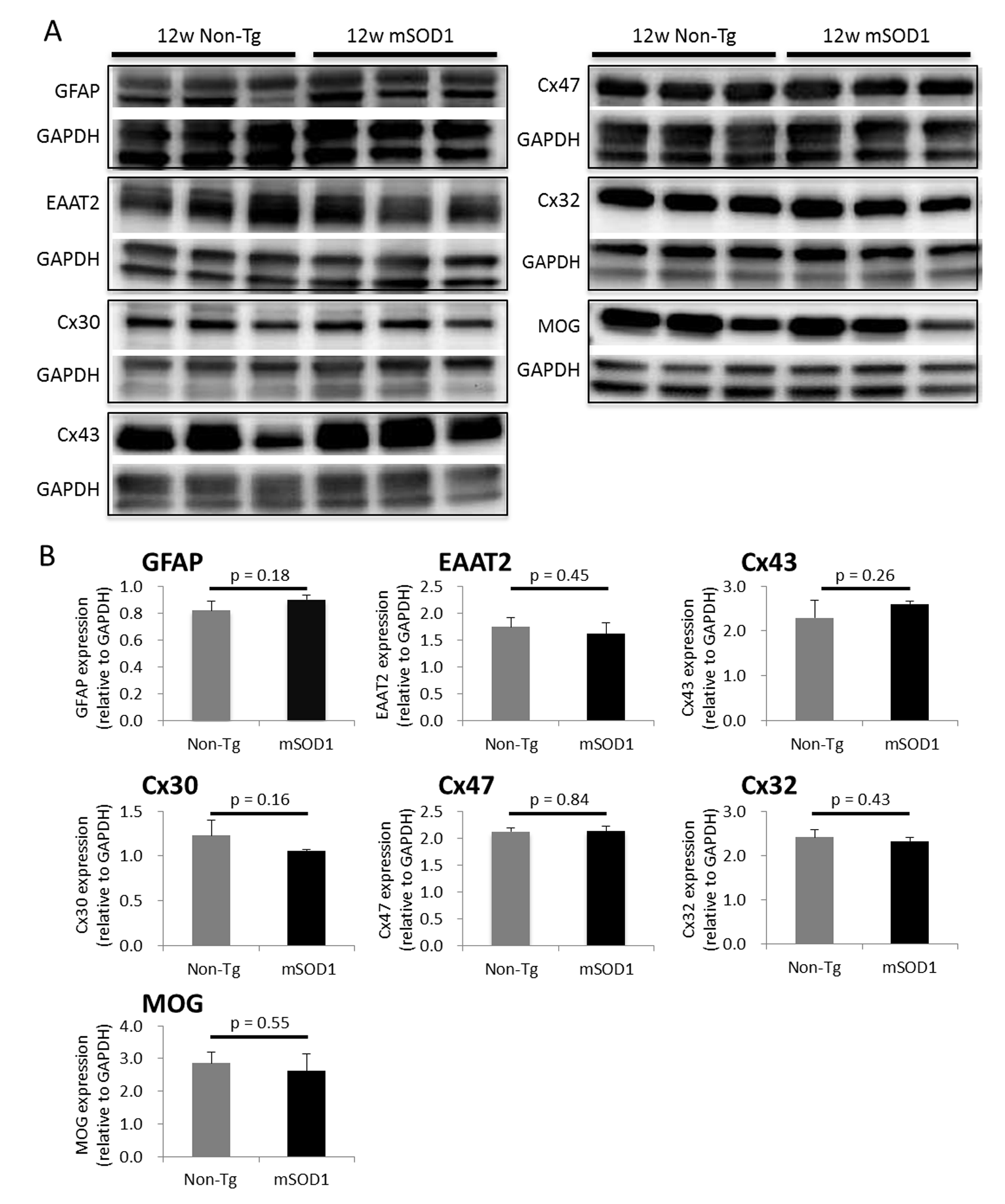

Supplement: Additional file 5: Figure S5 — Quantitative immunoblot analysis of Cxs in mSOD1-Tg mice and non-Tg mice at 12 weeks of age. (A) Representative images of GFAP, EAAT2, Cx43, Cx30, Cx47, Cx32 and MOG immunoblots obtained from mSOD1-Tg mice and non-Tg mice (n = 3 per group). GAPDH blots for loading controls are shown under each protein blot. (B) Results of quantitative analysis for each protein. There is no statistically significant difference between mSOD1-Tg and non-Tg mice for any markers of astrocytes or oligodendrocytes. [file 1742-2094-11-42-S5.tiff]
